# Evaluation of Two Pain Assessment Methods (Tactile and Air blast) for Comparison the Effectiveness of Nd:YAG Laser Therapy and Non-Laser Therapy on Dentin Hyper Sensitivity Treatment: A Systematic Review and Meta-analysis

**DOI:** 10.30476/dentjods.2022.93159.1698

**Published:** 2023-06-01

**Authors:** Zahra Baghani, Malihe Karrabi

**Affiliations:** 1 Dept. of Periodontics, Faculty of Dentistry, Sabzevar University of Medical Sciences, Sabzevar, Iran; 2 Dept. of Prosthodotics, Faculty of Dentistry Sabzevar University of Medical Sciences, Sabzevar, Iran

**Keywords:** Dentin Hypersensitivity, Nd:YAG Laser, Topical Desensitizing Agent, Air Blast Test, Tactile Test, Meta-analysis

## Abstract

**Statement of the Problem:**

Dentin hypersensitivity (DH) is a common irritating condition. A precise sensitive test for its assessment can greatly aid in appropriate treatment planning.

**Purpose:**

This meta-analysis aims to compare the air blast and tactile tests for assessment of the efficacy Nd:YAG laser therapy versus non-laser treatments for DH in short-term and long-term follow-ups.

**Materials and Method:**

For this review, an electronic search of the literature was carried out in three databases by two researchers for English articles published until March 10, 2021. Pooling of the data extracted from the selected articles was performed according to the PRISMA statement by the random-effect model. The mean difference (MD) and 95% confidence interval (CI) of pain score before the treatment onset and during the follow-up period according to the visual analog scale (VAS) were calculated. The level of heterogeneity was assessed by the I_2_ test, and a funnel plot was drawn to assess the publication bias of the reviewed studies.

**Results:**

Of 152 articles primarily retrieved, 9 randomized clinical trials (RCTs) using the air blast test and 4 RCTs using the tactile test were subjected to quantitative synthesis. In the short-term follow-up and immediately after treatment, the air blast test showed superiority of laser therapy compared with non-laser treatments (SMD: 0.55, 95% CI: 0.05-1.04, *p*= 0.03). However, this difference was not significant according to the tactile test (SMD: 0.48. 95% CI: 0.01-0.96, *p*= 0.06). In the long-term follow-up, the difference between laser therapy and non-laser modalities was not significant according to both air blast (SMD= -0.38, 95% CI: -1.43-0.67, *p*= 0.48) and tactile (SMD=0.0, 95% CI: -0.38-0.38, *p*= 0.99) tests.

**Conclusion:**

Comparison of laser therapy and non-laser modalities in the short-term reveal-ed higher sensitivity of the air blast test due to its mechanism of action compared with the tactile test. Further studies are required to interpret the results in the long-term follow-up.

## Introduction

Dentin hypersensitivity (DH) refers to a transient sharp pain in exposed dentin following external stimulation by thermal, electrical, mechanical, tactile, chemical, or osmotic stimuli, or evaporation. Pain due to DH cannot be attributed to any other reason. Cold is the most common trigger for DH [ 1 ]. 

Root exposure due to gingival resorption followed by periodontal disease is one of the most common causes of this problem. New evidence indicates that the mechanism of DH pain can be explained by combination of hydrodynamic theory and neural theory. This can be explained that because of the abovementioned stimuli, the movement of dentinal fluid changes the intrapulpal pressure around odontoblasts and their odontoblastic processes, which leads to stimulation of intradental myelinated A- β and A- δ fibers, resulting in generation of a transient sharp pain [ 2 - 3 ]. 

High prevalence and irritating nature of DH, and high risk of diagnostic errors can lead to its misdiagnosis, for instance with the symptoms of dental caries, and subsequent aggressive treatments. Air blast (evaporation), cold water, thermal and tactile stimuli, and subjective examination can be used for the assessment and identification of pain, and evaluation of the efficacy of different treatments for DH. Each test employs a specific stimulus for pain induction, such as mild air stream of air spray in air blast test, the tip of a probe in tactile test, and ice in thermal stimulation test [ 1 , 4 ]. Of the abovementioned tests, the air blast and tactile tests are more commonly used for the assessment of DH due to their physiological nature and reproducibility [ 5 ]. Nonetheless, selection of a precise and reliable test for assessment of the efficacy of treatments is a challenge for dental clinicians because in absence of a precise and sensitive test, accurate treatment planning would not be possible. Thus, selection of a reliable test is a fundamental step in assessment of treatment efficacy. The mechanism of action of these tests is based on the movement of dentinal fluid and stimulation of odontoblastic processes [ 6 ]. Thus, the efficacy of the available treatment modalities for DH such as topical desensitizing agents and laser therapy, which are based on sealing of dentinal tubules and reduction of the movement of dentinal fluid and stimulation of odontoblastic processes, can be well evaluated by these tests [ 7 ]. Of the available treatments for DH, topical desensitizing agents are the most affordable, widely accessible, and most frequently used modalities. However, their effects are short-term since the deposits sealing the tubules are removed over time due to the consumption of acidic foods and drinks, and tooth brushing [ 6 - 7 ]. Therefore, laser therapy with CO2, Er, Cr:YSGG, Er:YAG, Nd:YAG and GaAlAs lasers were suggested for treatment of DH. Depending on the type and wavelength of laser, laser therapy can have a success rate of 5.2% to 100% for treatment of DH [ 8 ]. The effectiveness of different lasers in reducing the diameter of dentin tubules has been proven [ 9 ]. Among them, Nd:YAG laser has shown the highest efficacy for resolution of DH due to the melting of hydroxyapatite crystals, fusing and re-solidification of dentin along with analgesic properties causing no damage to teeth structure [ 10 - 12 ]. However, a comprehensive treatment for DH has yet to be introduced. Substantial variations in selection of a precise test for assessment of DH, study designs, and frequency, duration and time interval of follow-up sessions are factors that need to be taken into account in review studies and meta-analyses on DH. Previous studies that used these tests to compare the efficacy of laser- and non-laser treatments for DH with different follow-up periods have reported controversial results [ 13 - 16 ], which can be due to the differences in pain assessment tests, assessment time points, variable study designs, and use of different scales. Some meta-analyses on different tests combined the results of randomized clinical trials (RCTs) to compare the efficacy of laser and non-laser treatment modalities based on visual analog scale (VAS) or visual rating scale (VRS) scores [ 11 , 17 ]. However, such studies have numerous methodological flaws such as absence of precise inclusion criteria, use of different teeth in different studies, various follow-up periods, different assessment scales (VAS or VRS), using different tests (tactile, air blast, or thermal stimulation), and high heterogeneity; all these factors can contribute to unreliable results. Some meta-analyses combined the results of different tests [ 11 , 18 ]; while the stimulation threshold might be variable for different tests because each test works based on a particular stimulus. 

A recent systematic review evaluated the efficacy of different laser types for treatment of DH in comparison with non-laser modalities and placebo by combining the results of different tests, and found no significant difference between laser and non-laser modalities. However, the best results were obtained when both types of treatments were combined. Moreover, among the tested laser types, Nd:YAG laser showed the highest efficacy [ 11 ]. This study showed that different tests are suitable for assessment of the efficacy of laser treatments. However, combining the results of different tests, not differentiating between different laser types and absence of long-term follow-ups necessitate further securitization of this topic. A recent meta-analysis evaluated the use of air blast test to compare the efficacy of Nd:YAG and diode lasers versus desensitizing agents for treatment of DH over different follow-up periods. However, they combined the results of the two laser types and did not assess the results in the long-term, which highlights the need for more comprehensive studies on this topic [ 17 ]. 

It appears that using an accurate and reliable test can increase the validity of RCT and systematic reviews to eliminate the ambiguities regarding the accuracy of the results of equivocal studies. Therefore, a comprehensive targeted study is required to find the most effective test for assessment of DH. This meta-analysis aims to compare two commonly used tests for assessment of DH namely the air blast and tactile tests to compare the efficacy of Nd:YAG laser therapy and non-laser modalities for treatment of DH in the short-term and long-term. 

## Materials and Method

This systematic review and meta-analysis was conducted in accordance with the PRISMA, Cochrane Collaboration, and Check Review checklists.

### Focused Clinical Question

What is the difference between the tactile and air blast pain assessment tests for evaluation of the efficacy of Nd:YAG laser and non-laser treatments for DH in the short-term and long-term follow-ups?

### Search Strategy

An electronic search of the literature was conducted in MEDLINE via PubMed, Cochrane and EMBASE, Scopus, and Science Direct databases by two researchers (ZB and MK) for English articles published until March 10, 2021 on the efficacy of Nd:YAG laser and non-laser treatments for DH during the follow-up sessions. The following MeSH and text words were used for the search of articles: Dentin sensitivity (MeSH) OR dentin hypersensitivity AND Nd:YAG Laser OR neodymium-doped yttrium aluminum garnet laser. 

### Eligibility Criteria

### Inclusion Criteria

Two reviewers (ZB and MK) evaluated the articles for the eligibility criteria. The inclusion criteria were as follows:

• *Participants:* Systemically healthy adults with DH

• *Intervention:* Nd:YAG laser with no limitation in power or management method

• *Comparison:* Use of a non-laser treatment modality such as toothpaste, gel, mouthwash, and so on

• *Outcome:* Assessment of DH during the follow-up sessions by air blast or tactile test using a VAS

• *Study design:* Only RCTs

### Exclusion Criteria

The following studies were excluded:

• *In vitro* and animal studies, review articles, and unpublished manuscripts

• Studies on patients with postoperative DH after procedures such as bleaching, periodontal treatment, and restorative procedures

• Use of VRS or SCASS for assessment of pain 

• Use of probe stimulation, cold water, or thermal stimuli to induce pain

• Not reporting baseline data in the study

• Full-text in a language other than English 

### Data Extraction

Two independent reviewers (ZB and MK) evaluated the selected studies and the following information was extracted including title of the study and the first author’s name, publication year, country, study design, number of participants, details of intervention and control groups, follow-up times, assessment methods, and the air blast and tactile test scores in both the intervention and control groups. Data were analyzed by two researchers and in case of disagreement a third reviewer was consulted. 

### Outcome Measurement

Any changes in the mean VAS pain score in air blast and tactile tests during the follow-up sessions compared with baseline in laser and non-laser groups were calculated.

### Risk of Bias and Assessment of the Quality of Evidence

Two masked reviewers conducted qualitative assessment of the methodology of the selected RCTs according to the Consolidated Standards of Reporting Trials. The following criteria were evaluated including random sequence generation, allocation concealment, blinding of participants and personnel, blinding of assessors, incomplete outcome data reporting, selective outcome reporting, and other sources of bias [ 18 - 20 ]. 

Comprehensive assessment of the risk of bias of studies was conducted according to the following criteria:

*High risk of bias:* studies that were rendered high-risk in at least one item 

*Unclear risk of bias:* Studies that had unclear risk of bias in one or more items

*Low risk:* Studies that were low risk in all items 

### Data Synthesis and Statistical Analyses

The mean VAS pain scores extracted from RCTs in air blast and tactile tests in different follow-up sessions were entered into a database. The heterogeneity of the studies was evaluated by the I_2_ test and P≤0.05 was considered statistically significant. Since the follow-up sessions had been conducted at different time points, subgroup analyses were performed according to different follow-up times to decrease heterogeneity. In addition, the effect size was calculated by using the mean difference (MD) and 95% confidence interval (CI), and the risk difference (95% CI) was also calculated for pooling of the results of each treatment group for pairwise data. For meta-analysis of the data and due to observation of heterogeneity in the MDs among the studies, random-effect models were used in Review Manager (RevMan) version 5.0 software. Moreover, for visual detection and quantitative analysis of the publication bias in each result, the funnel plot and trim and fill method were used in Additional Statistical Software Package (STATA version 16, STATA Corp., College Station, TX, USA). 

## Results

### Study Selection

The primary search of databases yielded 152 articles out of which, 81 were eliminated since they were duplicates. After reading the titles and abstracts of the remaining 71 articles, 42 were excluded since they did not meet the inclusion criteria. The full-text of the articles that their title and abstract met the eligibility criteria and those with non-informatory abstracts was read. Of the remaining 29 articles, 20 were excluded due to the following reasons:

1. Tactile or cold water test (2 articles) [ 21 - 22 ]
2. Not having a non-laser treatment group: (8 articles) [ 23 - 30 ]
3. Molar incisor hypomineralization (MIH) teeth: (1 article) [ 31 ]
4. Inaccurate data: (2 articles) [ 10 , 32 ]
5. Review articles and meta-analyses: (7 articles) [ 2 , 11 , 17 , 33 - 36 ]

**Figure 1:**
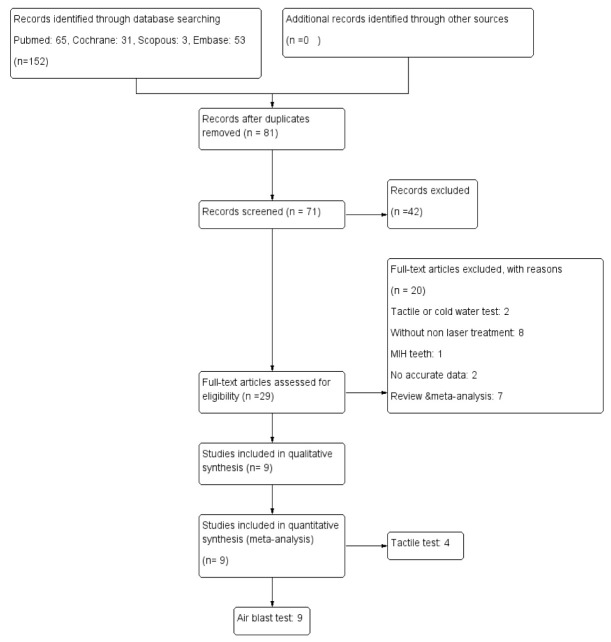
PRISMA 2009 flow diagram

Thus, in final quantitative analysis, 9 articles for the air blast test [ 4 , 13 - 14 , 37 - 43 ] and 4 articles for the tactile test [ 13 , 38 - 39 , 41 ] were extracted (Figure 1).

### Description of Studies

Nine selected RCTs had two arms comparing laser and non-laser treatment modalities. Accordingly, 606 patients were evaluated out of which, 302 were in the laser (test group), and 304 were in the non-laser (control) group. VAS was used in all studies to assess the level of pain at baseline, after the treatment, and during the follow-up sessions. Table 1 presents the main characteristics of RCTs evaluated in this study in brief. 

**Table 1:** Main characteristics of the reviewed studies

First author	Year	Country	Number of participants	Number of teeth	Test	Duration of air blast test	Intervention		Follow-up period
							Test group (laser treatment)	Control group (non-laser treatment)	
Kumar *et al*. [ 40 ]	2005	India	20	20 (10,10)	Air	1 sec	Nd:YAG laser: 30 mJ per pulse and 10 pulses per second by light painting for 2 minutes	Sodium fluoride varnish	Immediate
Kara *et al*. [ 14 ]	2009	Turkey	10	20 (10/10)	Air	Up to 10 sec	Nd:YAG laser with 2 W power output, 100 mJ energy, 20 Hz frequency, pulsed mode, for 60 seconds	Fluoride varnish	Immediate
									1 week
									2 weeks
									3 weeks
Abed *et al*. [ 15 ]	2011	Iran	40	80 (40/40)	Air	Not mentioned	Nd:YAG laser (10 Hz, 1 W, 60 s, two times)	Sensikin (containing potassium nitrate and sodium fluoride)	Immediate
									1 week
									1 month
									3 months
									6 months
Talesara *et al*. [ 42 ]	2014	India	40	80 (40/40)	Air	1sec	Nd:YAG laser: 1 W, 10 Hz, and 60 s two times	potassium binoxalate gel	Immediate
									3 months
									6 months
									9 months
Soares *et al*. [ 43 ]	2016	Brazil	23	33 (16,17)	Air	Up to 30 sec	Nd:YAG laser: 1 W and 10 Hz for 60 seconds	2% fluoride gel	Immediate
									1 week
Lopes *et al*. [ 13 ]	2017	Brazil	13	26 (13,13)	Air Tactile	3 sec	Nd:YAG laser: pulse duration of 120 μs, energy per pulse of 100 mJ, energy density of ≈85 J/cm^2^, contact mode, power of 1 W, and repetition rate of 10 Hz	Desensitizer agent (Gluma Desensitizer)	Immediate
									12 months
									18 months
Chebel *et al*. [ 16 ]	2018	Lebanon	27	54 (27/27)	Air Tactile	4 sec	Nd:YAG laser: 60 mJ (energy, 2 Hz (repetition rate), 0.64 W (power), and 100 mJ pulse energy (35.8 J/cm^2^)	Varnish containing casein phosphopeptide-amorphous calcium phosphate	1 week
									1 month
									3 months
									6 months
Maximiano *et al*. [ 39 ]	2019	Brazil	124	251 (124/127)	Air Tactile	3sec	Nd:YAG laser:1 W power, repetition rate of 10 Hz, 100 mJ energy, and 85 J/cm^2^ energy density.	Calcium sodium phosphosilicate prophylaxis paste	Immediate
									1 week
									1 month
Guo *et al*. [ 41 ]	2019	China	21 14(7,7)	78 (39/39)	Air Tactile	1 sec	Nd:YAG laser: (1064 nm) set at 30 mJ and 10 pps for 60 seconds	Dentin bonding agent	Immediate
									1 week
									1 month
									3 months

### Risk of Bias Assessment and Evidence Grading

**Figure 2:**
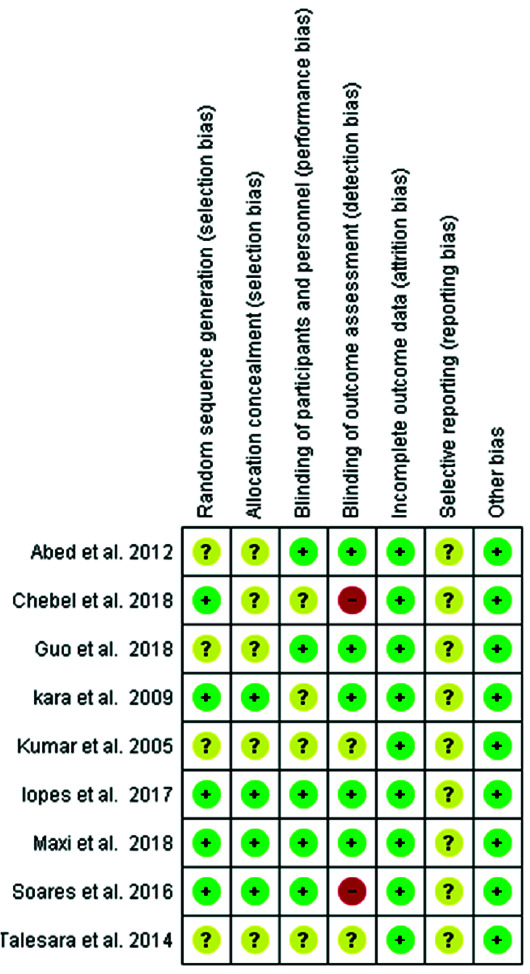
Quantitative analysis of publication bias evaluation

Figure 2 shows the result of quality assessment of the articles. None of the studies m*et al*l the evaluated criteria. Randomization of samples was questionable in four studies [ 15 , 40 - 42 ]. Allocation concealment was not disclosed in five studies [ 15 , 38 , 40 - 42 ]. Blinding of participants and personnel was not clear in four studies [ 14 , 16 , 40 , 42 ]. Blinding of outcome assessment was not disclosed in two studies [ 40 , 42 ] and was negative in two studies [ 38 , 43 ]. Thus, seven studies had unclear risk of bias while two studies had high risk of bias since the outcome assessment was not performed blindly.

### Outcomes of Meta-analysis

Since the results of tactile and air blast tests for pain assessment had been reported at different follow-up sessions in the reviewed studies, data analysis for the two tests was performed separately as subgroup analyses at different time points of immediate, one week, one month, 3 months, 6 months (For air blast test) and long-term. Due to the limited number of studies with long-term follow-ups, the long-term results for the air blast test included the results obtained at 9 to 18 months while the long-term results for the tactile test included the results obtained at 6 to 18 months. It should be noted that due to the limited number of studies, subgroup analysis based on the type of desensitizing agent or different laser settings was not performed.

### Pooled Follow-up Effect

For the air blast test, 604 patients were evaluated in nine studies with I_2_=85% heterogeneity. The results indicated that in general, laser therapy was significantly more effective than non-laser treatments (SMD=0.37, 95% CI: 0.12-0.61, *p*= 0.003) (Supplementary Figure 3). 

**Figure 3:**
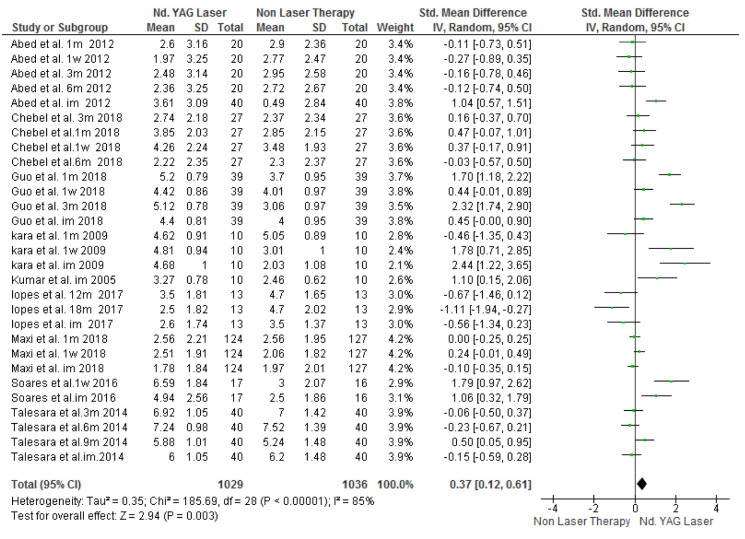
Forest plots of pooled visual analog scale (VAS) score change for Nd:YAG laser and topical desensitizing agents in air blast test (Rev Man)

For the tactile test, 370 patients were evaluated in four studies. Laser therapy was significantly more effective than non-laser treatments (SMD=0.53, 95% CI: 0.26-0.80, *p*= 0.0001) (Supplementary Figure 4).

**Figure 4:**
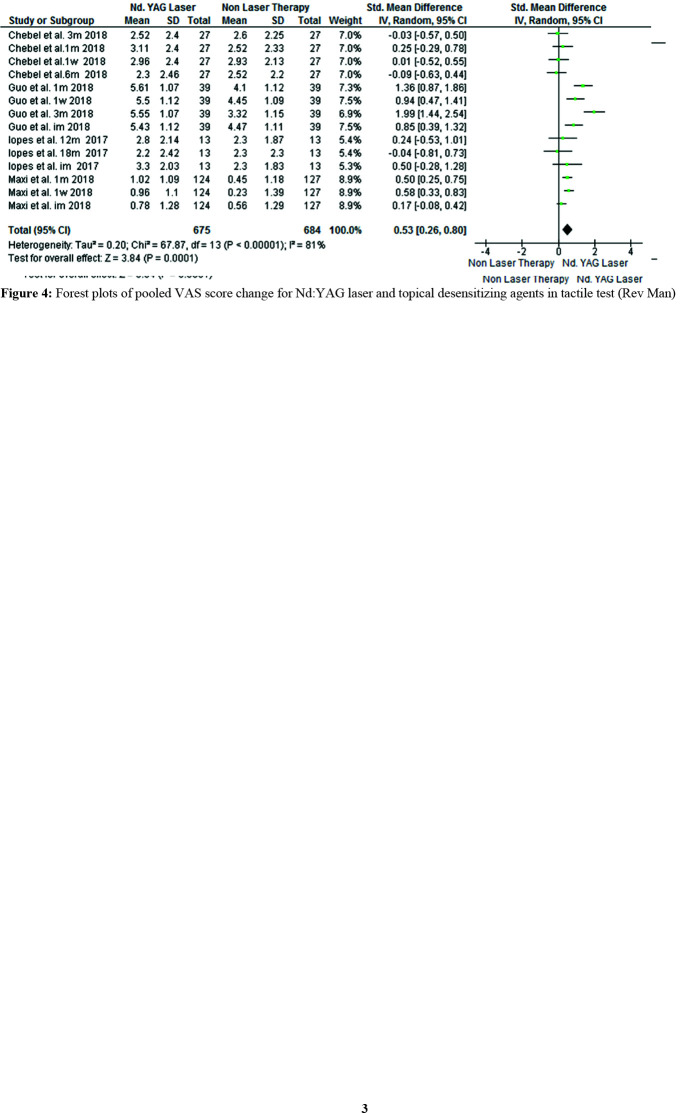
Forest plots of pooled visual analog scale (VAS) score change for Nd:YAG laser and topical desensitizing agents in tactile test (Rev Man)

### Immediate Effect

Assessment of the results immediately after treatment by the air blast test in 550 patients across 8 studied indicated that the success of laser therapy was significantly higher than that of non-laser treatments (SMD=0.55, 95% CI: 0.05-1.04, *p*= 0.03) (Supplementary Figure 5). However, tactile test conducted on 316 patients across three studies showed no significant difference in the efficacy of laser and non-laser treatments (SMD= 0.48, 95% CI:-0.01-0.96, *p*=0.06) (Supplementary Figure 6). 

**Figure 5:**
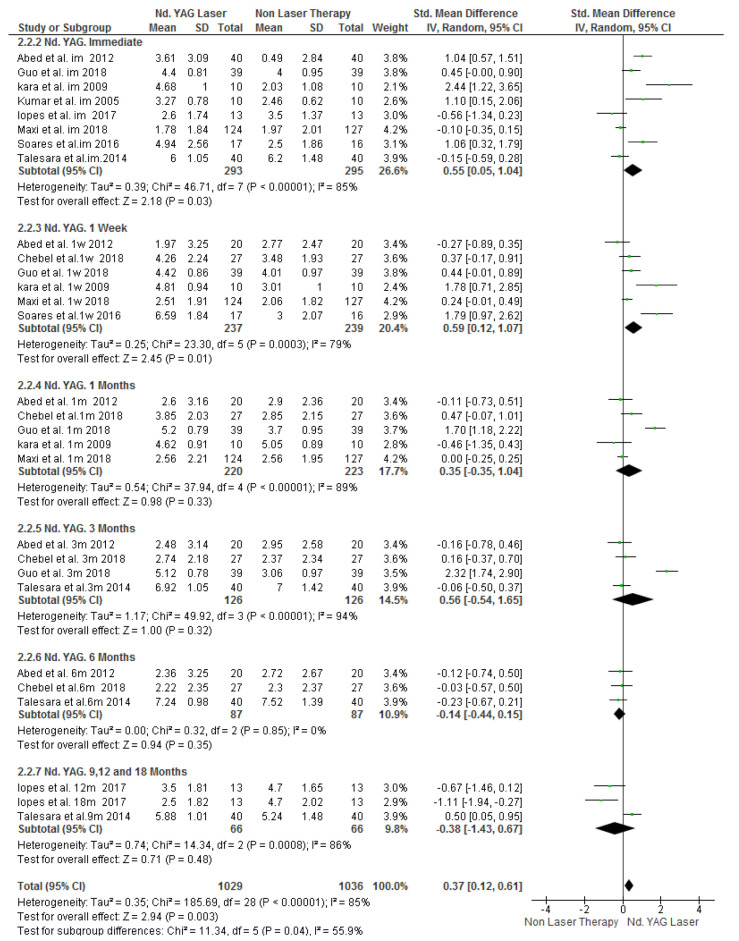
Forest plots of visual analog scale (VAS) score change for Nd:YAG laser and topical desensitizing agents in air blast test based on different follow up times (Rev Man)

**Figure 6:**
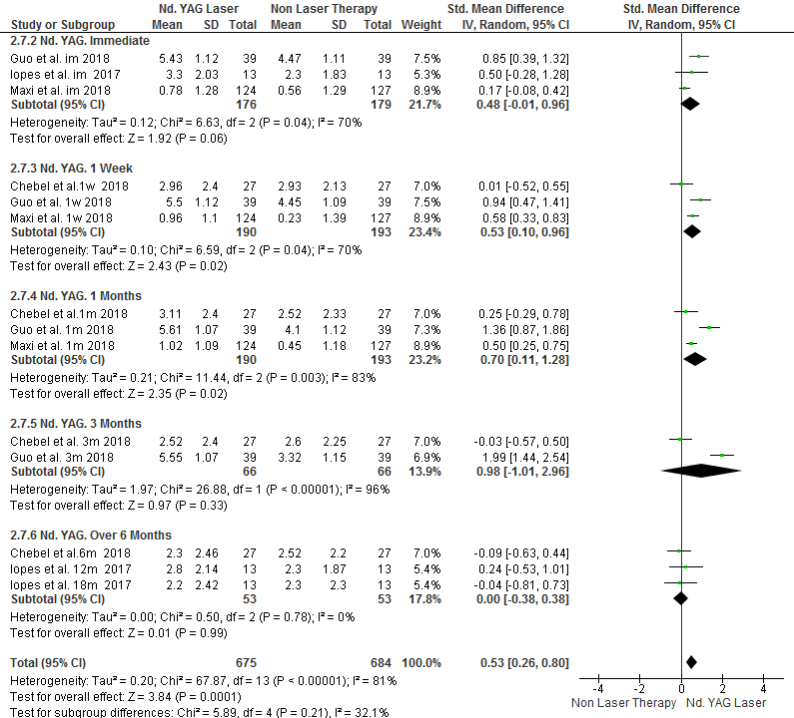
Forest plots of visual analog scale (VAS) score change for Nd:YAG laser and topical desensitizing agents in tactile test based on different follow up times (Rev Man)

### Effect at One Week

The results of the follow-up session at one week after treatment indicated the presence of a significant difference in favor of laser therapy in air blast test by assessing 478 patients across six studies (SMD=0.59, 95% CI:0.12-1.07, *p*= 0.01) (Supplementary Figure 5). The results of the tactile test on 344 patients across three studies indicated that laser therapy was significantly more effective than non-laser therapy (SMD=0.53, 95% CI:0.1-0.96, *p*= 0.02) (Supplementary Figure 6). 

### Effect at One Month

Data obtained regarding the air blast test were retrieved from 444 patients enrolled in five studies. The difference in the results at this time point was not significant (SMD: 0.35, 95% CI:-0.35-1.04, *p*= 0.33) (Supplementary Figure 5). However, the results of the tactile test in 344 patients across three studied revealed significant superiority of laser therapy (SMD:0.7, 95% CI:0.11-1.28, *p*= 0.02) (Supplementary Figure 6).

### Effect at Three Months

At three months, both treatment modalities were equally effective in reduction of DH such that the two tests (SMD=0.56, 95% CI:-0.54-1.65, *p*=0.32 for the air blast and SMD=0.98, 95% CI:-1.01-2.96, and *p*= 0.33 for the tactile test) found no significant difference in the efficacy of laser and non-laser treatment modalities (Supplementary Figures 5-6).

### Effect at Six Months

At this time point, only the results of air blast test were available, retrieved from 214 patients across three studies, which showed no significant difference in the efficacy of laser and non-laser treatments (SMD=-0.14, 95% CI:-0.44-0.15, *p*=0.35) (Supplementary Figure 5).

### Long-term Effect

The results at 9 to 18-month follow-ups in the air blast test were considered as the long-term results, and indicated no significant difference between the two treatment groups. It should be noted that this analysis was only performed on 132 patients across two studies (SMD=-0.38, 95% CI: -1.43-0.67, *p*= 0.48) (Supplementary Figure 7).

In the tactile test, the results at 6 to 18-month follow-ups were evaluated, which similar to the air blast test, showed no significant difference between the two treatment groups. This analysis was performed on 80 patients across two studies (SMD=0.0, 95% CI: -0.38-0.38, *p*=0.99) (Supplementary Figure 6).

### Publication Bias

In the present study, the tests to determine asymmetry for assessment of publication bias were performed on follow-up data immediately after treatment. Only one study by Chebel *et al*. [ 16 ] did not report the data immediately after treatment and due to the lack of high sensitivity between the data obtained at different time points, the data at one week after treatment was replaced (Supplementary Figure 7). Accordingly, the results of the funnel plot analysis of DH following laser and non-laser treatments did not show any asymmetry. Even in trim and fill analysis, no study was missed regarding this parameter (Supplementary Figure 8 (Stata Software) and Figure 9 (Rev Man Software. The regression asymmetry test did not show any publication bias either (Table 2).

**Figure 7:**
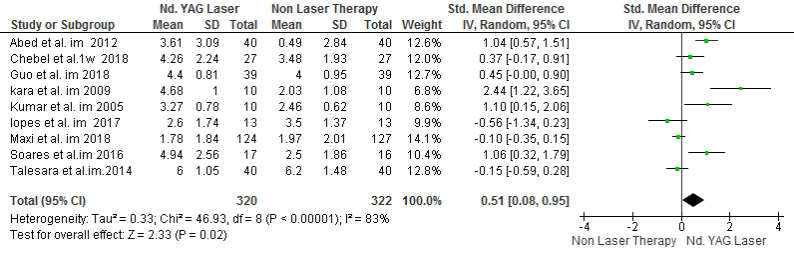
Forest plots of visual analog scale (VAS) score change for Nd:YAG laser and topical desensitizing agents in air blast test based on immediate follow up times (Rev Man)

**Figure 8:**
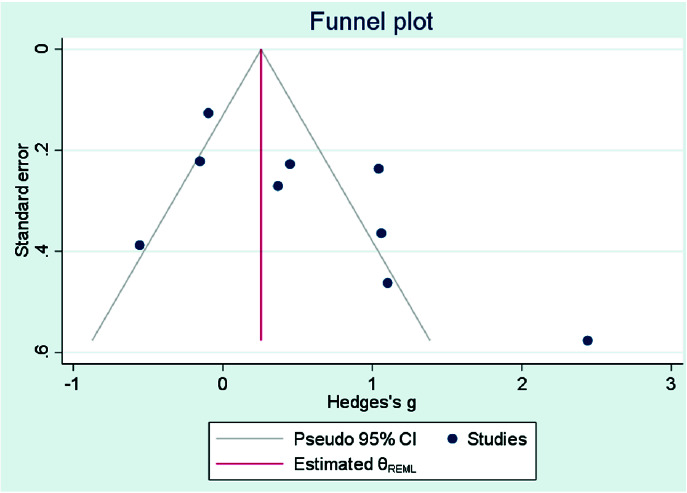
Forest plot and Funnel- plots for visual analog scale (VAS) score adjusted with Trim and Fill method (all included studies. Stata Software)

**Figure 9:**
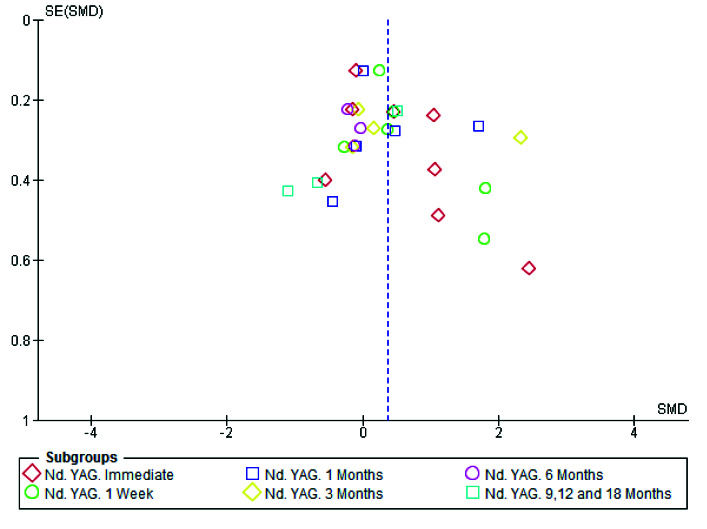
Forest plot and Funnel- plots for visual analog scale (VAS) score adjusted with Trim and Fill method (all included studies. Rev Man)

**Table 2:** Quantitative analysis for publication bias assessments

Original Meta-Analysis			Trim and Fill Analysis		
Outcome	MD (95% CI)	*p*	MD (95% CI)	Studies Trimmed/Total Studies	Egger Regression P
VAS score	0.51(0.08-0.95)	*p*<0.00001	0.51(0.08-0.95)	0	0.0264

MD: Mean difference

VAS: Visual analog scale

## Discussion

This meta-analysis compared the efficacy of two pain assessment tests namely the air blast and tactile tests for evaluation of the efficacy of Nd:YAG laser therapy and non-laser treatment modalities for treatment of DH at different time points. The results indicated that both tests yielded similar results and were effective for assessment of DH. However, it appears that the air blast test was more accurate and more sensitive for this purpose such that the air blast test with higher sensitivity showed significant superiority of laser therapy immediately and at one week after treatment. At other follow-up times, the results of both modalities were the same. However, the tactile test showed the superiority of laser therapy only at 1 week and 1 month, and the results immediately after treatment were not significant (similar to the long-term results), which was unexpected. This can be due to the different efficacy of treatments and the mechanism of action of the two pain assessment tests. 

In terms of efficacy of different treatment modalities, it should be mentioned that the mechanism of effect of all non-laser modalities evaluated in this study on DH is the same either through obstruction of the exposed dentinal tubules or by desensitization of the pulpal nerves [ 44 ]. Potassium-containing desensitizing agents prevent the conduction and transfer of nerve signals from the nerve terminal of dental pulp by inhibition of nerve cell repolarization, and do not obstruct the dentinal tubules [ 42 , 45 ]. Other desensitizing agents physically seal the dentinal tubules by deposition of crystals (first mechanism). However, evidence shows that the deposited crystals are washed away over time (approximately 6 months) due to different reasons such as wear caused by tooth brushing or consumption of citrus or acidic foods, resulting in recurrence of DH [ 42 ]. The mechanism of action of Nd:YAG laser for resolution of DH is different from the aforementioned mechanisms, and involves both analgesic effect on nerve cells and occlusion of dentinal tubules by melting of hydroxyapatite crystals at the same time. Of high-power lasers, Nd: YAG is the only type with analgesic effects, since it interferes with the function of sodium-potassium pump, changes the permeability of cell membrane, and alters the final part of the sensory axons. This analgesic effect may explain immediate pain resolution after laser therapy in patients treated with this laser as indicated by the air blast test. Obstruction of dentinal tubules due to melting of hydroxyapatite crystals would result in delayed effect of laser on DH such that after cooling of dentin surface following laser therapy, larger hydroxyapatite crystals are formed in the process of re-solidification. Thus, after recrystallization, a glazed nonporous surface is formed, which can partially or completely obstruct the dentinal tubules by up to 4-µm depth [ 42 , 46 ]. In addition, coagulation of proteins in dentinal fluid following laser therapy causes further occlusion of dentinal tubules, decreases their permeability, and consequently decreases the movement and flow of dentinal fluid [ 42 ]. Therefore, it appears that simultaneous analgesic effect and tubular obstruction by Nd:YAG laser is responsible for the considerable success rate of this modality and resolution of DH immediately after laser therapy and in short-term follow-ups compared with non-laser treatments since they do not have such a synergistic effect. However, the analgesic effect only lasts for a short period of time and after that, the mechanism of action of Nd:YAG laser in treatment of DH would only be the obstruction of dentinal tubules, which is similar to the mechanism of action of desensitizing agents. Also, due to the small diameter of laser fiber (0.2, 0.3, or 0.6mm), some open dentinal tubules may be missed in the process of scanning. Resultantly, the effect of laser remained significant only for one week after treatment according to the air blast test, and for one week and one month after treatment according to the tactile test. After this time, no significant difference was found in the efficacy of laser and non-laser treatments in reduction of DH.

Concerning the mechanism of action of the air blast and tactile tests, it appears that the difference in duration of effect of treatments might be due to the fact that in the air blast test, the movement of dentinal fluid occurs more intensely and in higher number of open dentinal tubules due to evaporation of dentinal fluid from the superficial parts of the exposed tubules. Consequently, greater stimulation occurs, which leads to pain generation. However, in the tactile test, evaporation of dentinal fluid does not occur. Moreover, due to the small cross-sectional area of the probe tip, a smaller number of open dentinal tubules are involved in contact of the probe tip and the exposed dentin surface. Therefore, both the intensity of movement of dentinal fluid, and number of stimulated dentinal tubules are lower in this test. Thus, the patient feels pain in higher degrees of dentin exposure. In other words, the tactile test requires more time to induce pain; sealing deposits should be worn enough for pain generation by probe stimulation. Thus, the air blast test shows a positive response sooner while the tactile test requires a longer time to show a positive response. This finding may explain the higher sensitivity of the air blast test compared with the tactile test, and points to the higher accuracy and reliability of the air blast test for assessment of dentin exposure especially in primary stages. To achieve more accurate results, the air syringe should be applied at a temperature of about 20°C (19-22°C) from a distance of 2 to 3mm from the tooth surface and in a position perpendicular to 90° with an intensity of 45 to 60 psi for 2 to 3 seconds [ 47 ]. Nonetheless, immediate results by the tactile test revealed no significant difference between the two treatment modalities, which appears to be due to the inclusion of the study by Lopes *et al*, [ 13 ] in their meta-analysis, and use of Gluma desensitizing agent in their study. Optimally high success rate of this desensitizing agent may be attributed to its constituents (5% glutaraldehyde and 35% hydroxyethyl methacrylate). The deposits formed by the reaction of glutaraldehyde with dentinal fluid proteins seal the dentinal tubules. In addition, these deposits cause polymerization of hydroxyethyl methacrylate, which can obstruct the tubules by the formation of resin tags up to 200-µm depth [ 48 ]. It appears that the formation of resin tags and high penetration depth of deposits into the dentinal tubules are responsible for the long-term durability of the results of treatment with Gluma. Such deposits provide a hermetic seal and have a lower risk of wash out, and wear by tooth brushing or the consumption of acidic foods and drinks. Since Lopes *et al*. [ 13 ] reported superior results for the desensitizing agent than laser in both the short-term and long-term follow-ups, inclusion of this article in the meta-analysis led to insignificant difference between the results of laser and non-laser treatment modalities immediately after the intervention as tested by the tactile test. It implies that the Gluma desensitizing agent is as effective as laser therapy for treatment of DH. However, further studies are required to confirm the efficacy of this desensitizing agent. Concerning the limitations of Nd:YAG lasers including irreversible injuries such as microcracks, carbonization of the tooth surface or an increase in intra-pulp temperature in power>1.5 W [ 49 ], this agent can be used as an alternative to laser therapy for DH. This is because in addition to its higher efficacy, it is more affordable and more easily accessible than laser, and its application is simpler than laser therapy for both the clinician and patient [ 13 ].

Furthermore, considering the positive and negative aspects of each method, the application of laser combined with non-laser treatments such as CPP-ACPF or Gluma desensitizer, besides their individual benefits, can overcome these limitations. It seems that their use in combination has additive effects in the treatment of DH. The effectiveness of this combined approach has been proven in various studies with the greatest effect in improving patients' pain [ 9 , 13 , 49 - 50 ]. 

In addition to the assessment of the efficacy of laser and non-laser treatment modalities for treatment of DH, this study evaluated the accuracy of pain assessment tests for evaluation of the efficacy of treatments. The results of the present meta-analysis confirmed the optimal efficacy of laser for resolution of DH pain. This finding was in agreement with the findings of previous studies that showed the superior efficacy of laser therapy compared with the negative control and placebo groups [ 33 , 51 ]. A recent study compared the efficacy of Nd:YAG and diode lasers with topical desensitizing agents, and showed the superior efficacy of laser therapy although the difference was not statistically significant [ 17 ]. Despite the attempts to create a meticulous methodology in previous meta-analyses, some factors were not addressed such as assessment of different laser types, equal follow-up intervals, separate evaluation of the results of different tests, and use of merely one scale for pain assessment (VAS or VRS). Although the abovementioned parameters were addressed in the present meta-analysis, it still had some limitations such as small sample size (both in number of studies and number of patients evaluated in each study), inadequate number of studies with long-term follow-ups, and inability to assess the efficacy of different desensitizing agents separately. For correct interpretation of results, further meta-analyses with more accurate designs are required on RCTs with larger sample sizes and longer follow-ups to compare laser therapy with only one particular type of desensitizing agent. 

## Conclusion

The current results indicated the comparable efficacy of Nd:YAG laser therapy and topical desensitizing agents for reduction of DH according to both air blast and tactile tests. Nonetheless, the air blast test had higher sensitivity for assessment of treatment results in the short-term compared with the tactile test due to its particular mechanism of action and more severe stimulation of a higher number of open dentinal tubules. However, interpretation of the results of the long-term follow-ups requires further investigations. 

## Conflict of Interest

The authors declare that they have no conflict of interest.
